# Effects of nicardipine and esmolol on regional renal oxygenation and hemodynamics during controlled hypotension in rhinoplasty: A prospective, randomized clinical trial

**DOI:** 10.1097/MD.0000000000049888

**Published:** 2026-07-24

**Authors:** Kamil Cintan, Nureddin Yüzkat, Canser Yilmaz Demir, Yunus Emre Tunçdemir

**Affiliations:** a Department of Anesthesiology and Reanimation, Van Regional Training and Research Hospital, Van, Turkey; b Department of Anesthesiology and Reanimation, Van Yuzuncu Yil University School of Medicine, Van, Turkey; c Department of Plastic and Reconstructive Surgery, Van Yuzuncu Yil University School of Medicine, Van, Turkey; d Department of Algology, Van Regional Training and Research Hospital, Van, Turkey.

**Keywords:** controlled hypotension, esmolol, nicardipine, regional renal oxygenation, rhinoplasty

## Abstract

**Background:**

Achieving a bloodless surgical field during rhinoplasty is critical, yet the physiological impact of controlled hypotension (CH) on renal oxygenation remains a concern. This study evaluated the effects of nicardipine and esmolol on regional renal oxygenation and hemodynamics during controlled hypotension in patients undergoing rhinoplasty.

**Methods:**

After ethics approval and trial registration (ClinicalTrials.gov: NCT05430724), 80 adults with ASA I-II status undergoing elective rhinoplasty at a single university hospital were enrolled. Patients were assigned to group N (nicardipine, n = 40) or group E (esmolol, n = 40). Target mean arterial pressure (MAP) was 55 to 65 mm Hg. The primary outcomes were regional renal oxygenation (rSO_2_) measured using near-infrared spectroscopy and hemodynamics. Secondary outcomes included opioid requirements, renal function tests, and assessor-blinded surgical satisfaction.

**Results:**

All 80 randomized patients completed the trial and were included in the final analysis. Repeated-measures analysis showed significant time-dependent changes in MAP and renal rSO_2_ values, with significant time × group interactions for MAP, right renal rSO_2_, and left renal rSO_2_. No prespecified renal desaturation events, defined as a ≥20% decrease from baseline rSO_2_, occurred in either group. Additional opioid use was significantly higher in group N (*P* = .001), whereas surgical satisfaction was more favorable in group E (*P* = .001). Postoperative creatinine and urea values remained within normal limits in both groups.

**Conclusion:**

In this short-duration rhinoplasty model, esmolol was associated with more favorable hemodynamic control, reduced opioid requirement, and higher surgical satisfaction compared with nicardipine. Although nicardipine was associated with higher regional renal rSO_2_ values, this finding should be interpreted as an exploratory physiological observation rather than evidence of functional renal protection. No external funding was received for this trial.

## 1. Introduction

Controlled hypotension (CH) is a widely used anesthetic technique in various surgical procedures, including rhinoplasty, to reduce intraoperative bleeding and improve surgical field visibility.^[1-3]^ CH typically targets a mean arterial pressure of 50 to 65 mm Hg or a 20% to 30% reduction from baseline while preserving end-organ perfusion.^[1,4,5]^ In rhinoplasty, where even minor bleeding may impair visualization of the operative field, CH has been associated with improved surgical conditions and surgeon satisfaction.^[2,6]^

Several pharmacological agents, including nicardipine, esmolol, dexmedetomidine, remifentanil, and nitroglycerin, have been used to achieve CH.^[3,6-8]^ However, these agents differ substantially in their hemodynamic mechanisms and potential effects on organ perfusion. Nicardipine, a dihydropyridine calcium channel blocker, lowers arterial pressure primarily through systemic vasodilation and reduction of vascular resistance.^[9]^ In contrast, esmolol, an ultra-short-acting selective β1-adrenergic antagonist, reduces blood pressure predominantly by decreasing heart rate and cardiac output.^[10-12]^

These distinct physiological mechanisms may differentially influence regional blood flow and tissue oxygen delivery, particularly in perfusion-sensitive organs such as the kidneys.

The kidneys are highly susceptible to reductions in perfusion pressure, and intraoperative hypotension has been associated with postoperative acute kidney injury (AKI), especially when hypotension is severe or prolonged.^[4,5]^ Although CH effectively improves surgical field quality, its impact on regional renal oxygenation remains insufficiently characterized, particularly when different hypotensive agents are compared. Furthermore, conventional renal biomarkers such as serum creatinine and urea are relatively insensitive for detecting early or subclinical renal hypoperfusion.

Near-infrared spectroscopy (NIRS) enables continuous, noninvasive monitoring of regional tissue oxygen saturation and may provide real-time information regarding renal oxygenation during CH.^[13-16]^ Recent evidence suggests that renal desaturation measured using NIRS may be associated with postoperative AKI; however, clinically meaningful thresholds and predictive reliability remain uncertain.^[17]^ Consequently, comparative evaluation of different hypotensive agents using renal NIRS monitoring may provide clinically relevant insights into the physiological effects of CH on renal oxygenation.

Therefore, this study aimed to compare nicardipine and esmolol in terms of hemodynamic control and regional renal oxygenation during CH in patients undergoing rhinoplasty.

## 2. Materials and methods

### 2.1. Ethics approval and registration

This study was conducted in accordance with the Declaration of Helsinki and CONSORT (Consolidated Standards of Reporting Trials) guidelines. Ethical approval was obtained from the Van Yüzüncü Yil University Clinical Research Ethics Committee (Decision No. 2019/03, Date: November 20, 2019). The trial was registered at ClinicalTrials.gov (NCT05430724). The study was carried out at Dursun Odabaş Medical Center at Van Yüzüncü Yil University between 12 May and August 31, 2022.

The inclusion and exclusion criteria for patients were as follows: 18 to 60 years of age, classified as ASA I–II, who underwent elective rhinoplasty surgery, received CH, had a body mass index (BMI) between 18 and 25 kg/m^2^, were informed about the study and provided written consent, and were included in the study. Patients classified as ASA III and IV; those with a history of revision rhinoplasty surgery; known allergies to the study drugs; hypertension; anticoagulant drug use; pregnancy; kidney failure; bleeding diathesis; fever; hypothermia; skin–kidney distance >4 cm, and those taking antibiotics, anticonvulsants, and antiarrhythmics were excluded from the study.

### 2.2. Randomization

Eligible patients were randomly assigned to 2 groups using the sealed-envelope method. Patients who received esmolol were designated as group E (n = 40), and those who received nicardipine were designated as group N (n = 40).

### 2.3. Preoperative preparation

Premedication was administered with 0.02 to 0.03 mg/kg midazolam before the patient was taken to the operating room. Vascular access was obtained from a peripheral vein via a 20 G cannula, and hydration was initiated with 5 to 10 mL/kg 0.9% NaCl fluid. Electrocardiogram (ECG), SpO_2_, heart rate (HR), and noninvasive blood pressure monitoring were performed.

Before anesthesia induction, bedside ultrasonographic assessment was performed to evaluate the skin-to-kidney distance, and only patients with a depth ≤4 cm were included because of the known penetration depth limitations of NIRS monitoring. Bilateral NIRS sensors (INVOS Adult Sensor, Medtronic, Minneapolis) were then placed over the T_10_–L_2_ flank regions corresponding to the renal projection areas. Baseline measurements were obtained before induction, and renal NIRS values were recorded at 5-minute intervals during the intraoperative period.

### 2.4. Induction and maintenance of anesthesia

All patients were preoxygenated with 100% O_2_ for 3 minutes before induction. In all patients, lidocaine (1–1.5 mg/kg), propofol (2–2.5 mg/kg), fentanyl (2–2.5 mcg/kg), and rocuronium (0.6 mg/kg) were administered intravenously during induction. The patients were then intubated and provided with respiratory support and mechanical ventilation. During anesthesia maintenance, ventilation was provided with a mixture of 40% oxygen and 60% dry air, a tidal volume of 6 to 8 mL/kg, and a respiratory rate of 12 to 16 breaths per minute, with a flow rate of 2 L/min. Desflurane was used as the inhalational anesthetic during maintenance of anesthesia. Patients were continuously monitored hemodynamically throughout the surgery. End-tidal CO_2_ values were maintained between 30 and 35 mm Hg.

### 2.5. Procedure for controlled hypotension

In both groups, the target mean arterial pressure (MAP) was set at 55 to 65 mm Hg. Prior to the commencement of surgery, group N was administered nicardipine infusion at 5 to 15 mg/h, while group E received esmolol infusion at 50 to 300 µg/kg/min. The infusion rates of nicardipine and esmolol were titrated in groups N and E, respectively, to achieve and maintain the predetermined target MAP. The infusion rate was increased if the MAP exceeded the intended level for > 5 minutes. During the operation, an additional anesthetic drug, remifentanil, was administered intravenously at a dose of 0.5 to 1 mcg/kg if the target MAP was not achieved. If necessary, 10 mg of rocuronium was used as a muscle relaxant. If the MAP fell below the desired value for more than 5 minutes, the dose of anesthetic medications was decreased and an intravenous bolus of fluid was administered. If this amount was insufficient, 5 mg ephedrine was administered intravenously. Bradycardia was defined as a heart rate of <45 beats per minute for >2 minutes, and the drug dose was reduced in both groups. If the response was insufficient, 1 mg atropine was administered intravenously. At the end of the surgical procedure, nicardipine and esmolol, prescribed for CH, were discontinued.

A decrease in regional renal NIRS values of more than 20% from baseline at any time was considered indicative of impaired renal oxygenation. To optimize the renal NIRS values, the anesthetic agent dosage was decreased, the MAP value was increased, the FiO_2_ was increased, and the end-tidal CO_2_ value was increased.

### 2.6. Measurements

Patient demographic data, BMI, and smoking status were also recorded. Noninvasive blood pressure, HR, SpO_2_, renal NIRS (right/left rSO_2_), and opioid and muscle relaxant doses and quantities were recorded every 5 minutes at baseline, intraoperatively, and post-induction. The duration of the operation and anesthesia, as well as the preoperative and postoperative urea and creatinine levels, were also recorded. The total volume of the postoperative hemorrhage was measured and recorded. Immediately after the procedure, postoperative surgical satisfaction regarding the quality of the surgical field was assessed by the operating surgeon, who was blinded to the group allocation, and the assessments were recorded by an independent investigator who was blinded to the group allocation using a 5-point Likert scale (1 = not at all satisfied, 5 = very satisfied).

### 2.7. Statistical analysis

The sample size was determined prior to patient enrollment to detect a clinically meaningful difference in regional renal tissue oxygenation measured by near-infrared spectroscopy (NIRS). Because validated renal desaturation thresholds during controlled hypotension have not yet been clearly established, the sample size estimation and renal desaturation criteria were based on previously published adult renal NIRS studies. Choi et al^[18]^ reported a baseline renal regional oxygen saturation (rSO_2_) value of 80.0 ± 9.1% and evaluated clinically relevant renal desaturation using both absolute thresholds and proportional decreases from baseline, including 10%, 15%, 20%, 25%, and 30% reductions in renal rSO_2_. In addition, Tholén et al^[19]^ demonstrated a significant correlation between renal rSO_2_ and invasively measured renal venous oxygen saturation in adult cardiac surgery patients and reported a measurement variability of approximately 6% to 7% SD for renal oxygenation values.

Based on these renal NIRS data, a 4.5% absolute difference in renal rSO_2_ between the groups was considered clinically meaningful. Assuming a standard deviation of approximately 6%, a 2-sided α error of.05, and a statistical power of 80%, the minimum required sample size was calculated as 29 patients per group. To compensate for possible dropouts and incomplete measurements, 40 patients were enrolled in each group.

Renal desaturation was additionally evaluated as a ≥20% decrease from baseline renal rSO_2_, consistent with previously published renal NIRS methodologies using proportional decreases from baseline.^[18]^

For repeated intraoperative measurements of MAP and renal rSO_2_, repeated-measures analysis of variance was performed to evaluate the effects of time, group, and the time × group interaction. Mauchly’s test was used to assess the sphericity assumption. When sphericity was violated, Greenhouse–Geisser correction was applied. Effect sizes were reported as partial eta squared (partial η^2^).

Descriptive statistics (mean, standard deviation [SD], median, minimum, maximum, frequency, and percentage) were used to summarize the data. The Kolmogorov–Smirnov test was used to assess normality of distribution. Independent samples *t* tests and Mann–Whitney *U* tests were used for comparisons of quantitative independent variables, whereas the Wilcoxon test was used for paired quantitative variables. Categorical variables were analyzed using the chi-square test or Fisher exact test when appropriate. Between-group comparisons at individual time points were performed without formal adjustment for multiple comparisons; therefore, time-dependent *P* values were considered nominal and should be interpreted as exploratory. Statistical analyses were performed using SPSS 26.0 (IBM Corp., Armonk).

## 3. Results

Initially, 87 patients were enrolled in the study; however, 7 were subsequently excluded (five underwent revision rhinoplasty surgery and 2 declined to participate). Thus, 80 patients were included in the final analysis, with 40 patients in each group.

### 3.1. Baseline characteristics and operative data

Baseline demographic characteristics did not differ significantly between the groups (*P* > .05). However, intraoperative bleeding volume was significantly greater in group N (*P* = .001; Table 1).

**Table 1:** Baseline characteristics and operative data.

		Group E (n = 40)		Group N (n = 40)		*P*
		Mean ± SD/n (%)	Median	Mean ± SD/n (%)	Median	
Age (yr)		26.6 ± 5.6	26.0	26.4 ± 6.0	25.5	.747*
Gender	F	26 (65%)		27 (67.5%)		.813†
	M	14 (35%)		13 (32.5%)		
Height (cm)		170.6 ± 8.7	169.5	166.6 ± 8.2	165.0	.052†
Weight (kg)		66.3 ± 12.9	63.0	65.1 ± 12.5	64.5	.870†
BMI (kg/m^2^)		22.7 ± 3.3	21.7	23.4 ± 3.5	22.9	*.346* *
ASA	I	24 (60%)		25 (62.5%)		*.818* ‡
	II	16 (40%)		15 (37.5%)		
Smoking	(−)	25 (62.5%)		29 (72.5%)		*.340* ‡
	(+)	15 (37.5%)		11 (27.5%)		
Amount of intraoperative bleeding (mL)		45.3 ± 16.0	40.0	107.8 ± 47.3	100.0	*.001* †
Duration of surgery (min)		62.37 ± 2.81	63	61.87 ± 2.86	61	*.441* †

ASA = American Society of Anesthesiologists, BMI = body mass index, SD= standard deviation.

* Independent sample *t* test.

† Mann–Whitney *U* test.

‡ Chi-square test.

### 3.2. Hemodynamic and regional renal oxygenation trends

Repeated-measures ANOVA was performed to evaluate the effects of time, group, and the time × group interaction for MAP, right renal rSO_2_, and left renal rSO_2_ values. Greenhouse–Geisser-corrected results were reported because the sphericity assumption was violated (Table 2).

**Table 2:** Repeated-measures ANOVA results for MAP and renal rSO_2_ values.

Variable	Effect	*F*	df	*P* value	Partial η^2^
MAP	Time	99.82	5.93, 462.63	<.001	0.561
	Group	2.10	1, 78	.151	0.026
	Time × group	7.77	5.93, 462.63	<.001	0.091
Right renal rSO_2_	Time	12.07	3.67, 285.94	<.001	0.134
	Group	2.77	1, 78	.100	0.034
	Time × group	3.19	3.67, 285.94	.017	0.039
Left renal rSO_2_	Time	13.35	3.77, 293.94	<.001	0.146
	Group	9.00	1, 78	.004	0.103
	Time × group	4.08	3.77, 293.94	.004	0.050

Greenhouse–Geisser-corrected repeated-measures ANOVA results are shown.

ANOVA = analysis of variance, df = degrees of freedom, MAP = mean arterial pressure, partial η^2^ = partial eta squared, rSO_2_ = regional oxygen saturation.

For MAP, repeated-measures ANOVA demonstrated a significant effect of time (*F*(5.93, 462.63) = 99.82, *P* < .001, partial η^2^ = 0.561), indicating that MAP values changed significantly during the intraoperative period. The overall group effect was not significant (*F*(1, 78) = 2.10, *P* = .151, partial η^2^ = 0.026). However, a significant time × group interaction was observed (*F*(5.93, 462.63) = 7.77, *P* < .001, partial η^2^ = 0.091), suggesting that the temporal MAP pattern differed significantly between the groups.

For right renal rSO_2_, repeated-measures ANOVA demonstrated a significant effect of time (*F*(3.67, 285.94) = 12.07, *P* < .001, partial η^2^ = 0.134), indicating that right renal rSO_2_ values changed significantly over time. The overall group effect was not significant (*F*(1, 78) = 2.77, *P* = .100, partial η^2^ = 0.034). However, a significant time × group interaction was observed (*F*(3.67, 285.94) = 3.19, *P* = .017, partial η^2^ = 0.039), suggesting that the temporal pattern of right renal rSO_2_ differed significantly between the groups.

For left renal rSO_2_, repeated-measures ANOVA demonstrated a significant effect of time (*F*(3.77, 293.94) = 13.35, *P* < .001, partial η^2^ = 0.146), a significant group effect (*F*(1, 78) = 9.00, *P* = .004, partial η^2^ = 0.103), and a significant time × group interaction (*F*(3.77, 293.94) = 4.08, *P* = .004, partial η^2^ = 0.050). These findings indicate that left renal rSO_2_ values changed over time, were overall higher in Group N, and followed a different temporal pattern between groups.

No patient in either group experienced the prespecified renal desaturation event, defined as a ≥20% decrease from baseline renal rSO_2_ at any intraoperative time point. Exploratory unadjusted time-point comparisons are presented in Tables 3–5 and should be interpreted as descriptive secondary findings rather than confirmatory evidence of individual time-point differences.

**Table 3:** Exploratory time-point comparisons of intraoperative MAP values.

Time	Group E (n = 40)		Group N (n = 40)		*P*
	Mean ± SD	Median	Mean ± SD	Median	
0 min	93.8 ± 11.9	92.5	91.7 ± 9.8	90.5	.414*
5 min	74.2 ± 10.0	72.5	80.3 ± 16.6	79.3	***.049*** *
10 min	70.7 ± 7.1	70.2	64.4 ± 11.6	61.2	**.004** *
15 min	66.6 ± 6.1	66.5	60.5 ± 11.2	58.7	**.001** *
20 min	63.3 ± 4.3	63.8	61.8 ± 9.5	58.0	.367*
25 min	62.1 ± 5.4	63.0	61.9 ± 11.3	58.5	*.913* *
30 min	60.7 ± 7.9	60.8	60.9 ± 10.8	57.7	*.941* *
35 min	59.5 ± 6.4	59.0	60.8 ± 11.2	56.5	*.526* *
40 min	59.5 ± 7.3	59.0	62.8 ± 11.6	60.5	*.416* *
45 min	57.7 ± 7.5	58.2	63.6 ± 10.9	61.3	***.014*** *
50 min	57.7 ± 9.1	56.0	65.7 ± 12.3	64.2	***.003*** *
55 min	57.2 ± 8.9	55.3	66.4 ± 12.9	63.8	***.001*** *
60 min	57.1 ± 9.4	55.3	67.8 ± 15.9	66.7	***.003*** *

0th min: Baseline value.

Bold values indicate statistically significant results (*P* < .05).

SD = standard deviation.

* Mann–Whitney *U* test.

**Table 4:** Exploratory time-point comparisons of right renal rSO_2_ values.

Time	Group E (n = 40)		Group N (n = 40)		*P*
	Mean ± SD	Median	Mean ± SD	Median	
0 min	84.9 ± 7.0	84.5	84.2 ± 6.9	85.0	.572*
5 min	87.0 ± 7.0	89.0	88.5 ± 5.5	90.0	*.445* *
10 min	85.2 ± 7.9	86.0	88.1 ± 6.4	90.0	.096*
15 min	84.9 ± 8.3	86.5	88.0 ± 6.3	89.5	.119*
20 min	84.6 ± 8.0	85.5	87.6 ± 6.0	88.5	.138*
25 min	84.1 ± 8.3	86.0	87.8 ± 6.1	88.5	***.048*** *
30 min	84.2 ± 8.2	86.0	88.0 ± 6.5	90.0	***.040*** *
35 min	84.0 ± 7.7	85.5	87.2 ± 6.6	89.0	***.046*** *
40 min	83.6 ± 7.3	84.5	86.3 ± 6.9	87.5	***.048*** *
45 min	83.3 ± 7.3	85.0	85.8 ± 7.1	89.0	*.125* *
50 min	83.3 ± 7.2	84.0	85.6 ± 7.4	87.5	*.148* *
55 min	83.2 ± 7.3	84.0	86.3 ± 6.6	88.0	*.056* *
60 min	83.2 ± 7.3	84.0	84.6 ± 7.7	85.5	*.566* *

0th min: Baseline value; renal rSO_2_: renal near-infrared spectroscopy.

Bold values indicate statistically significant results (*P* < .05).

rSO_2_ = regional oxygen saturation, SD = standard deviation.

* Mann–Whitney *U* test.

**Table 5:** Exploratory time-point comparisons of left renal rSO_2_ values.

Time	Group E (n = 40)		Group N (n = 40)		*P*
	Mean ± SD	Median	Mean ± SD	Median	
0 min	83.6 ± 7.8	85.5	84.2 ± 8.2	85.5	.873*
5 min	85.7 ± 7.9	88.5	89.5 ± 6.0	91.0	*.084* *
10 min	85.2 ± 7.9	87.5	90.2 ± 5.8	92.0	**.004** *
15 min	85.0 ± 8.5	86.5	89.9 ± 5.7	92.0	**.007** *
20 min	84.5 ± 8.6	86.5	90.0 ± 5.5	91.5	**.003** *
25 min	84.3 ± 8.7	85.0	90.2 ± 5.5	92.0	***.001*** *
30 min	83.3 ± 9.1	84.5	90.3 ± 5.3	91.5	***.001*** *
35 min	83.3 ± 8.8	83.5	89.0 ± 6.0	90.0	***.002*** *
40 min	82.9 ± 8.9	83.5	88.6 ± 6.4	90.0	***.003*** *
45 min	83.0 ± 8.3	84.0	87.7 ± 7.1	89.0	***.009*** *
50 min	82.7 ± 8.5	83.5	87.1 ± 7.3	88.0	***.016*** *
55 min	82.4 ± 8.7	83.5	87.1 ± 8.1	90.0	***.014*** *
60 min	83.6 ± 8.1	85.0	83.1 ± 9.5	85.0	*.880* *

0th min: Baseline value; renal rSO_2_: renal near-infrared spectroscopy.

Bold values indicate statistically significant results (*P* < .05).

rSO_2_ = regional oxygen saturation, SD = standard deviation.

* Mann–Whitney *U* test.

### 3.3. Renal function tests

There was no significant difference in the preoperative or postoperative urea (*P* = .15 and *P* = .08, respectively) or creatinine (*P* = .40 and *P* = .58, respectively) values between the groups (*P > *.05). However, the postoperative creatinine values were significantly lower than the preoperative values (*P* < .05; Table 6).

**Table 6:** Analysis of preoperative and postoperative urea and creatinine values.

	Group E (n = 40)		Group N (n = 40)		*P*
	Mean ± SD	Median	Mean ± SD	Median	
Creatinine					
Preoperative	0.77 ± 0.11	0.75	0.76 ± 0.13	0.73	*.399* *
Postoperative	0.72 ± 0.14	0.70	0.74 ± 0.14	0.72	*.583* *
In-group exchange (*P*)	***.001*** †		***.004*** †		
Urea					
Preoperative	27.3 ± 6.2	29.0	25.2 ± 7.0	23.5	*.149* *
Postoperative	27.5 ± 6.8	28.0	24.7 ± 7.7	23.5	*.083* *
In-group exchange (*P*)	.925†		.492†		

Bold values indicate statistically significant results (*P* < .05).

SD = standard deviation.

* Mann–Whitney *U* test.

† Wilcoxon test.

### 3.4. Additional drug requirements and surgical satisfaction

Intraoperative additional opioid use was significantly greater in group N than in group E (87.5% vs 20.0%, *P = *.001). In group N, 13 patients received 1 additional opioid dose, 13 received 2 doses, 8 received 3 doses, and 1 received 4 doses. In contrast, only 8 patients in group E received additional opioids, and none required 3 or more doses. There was no significant difference in intraoperative muscle relaxant use between the groups (*P* = .49).

Surgical satisfaction levels were more favorable in group E than in group N (*P* = .001; Table 7). The *P* value represents the overall comparison of the distribution of surgical satisfaction levels between groups using the chi-square test, rather than a pairwise comparison of individual satisfaction levels.

**Table 7:** Surgical satisfaction levels.

Surgical satisfaction levels	Group E (n = 40)	Group N (n = 40)	*P*
Median (min–max)	4 (2–5)	3 (1–5)	***.001*** *
Very satisfied, n (%)	4 (10.0)	2 (5.0)	
Satisfied, n (%)	22 (55.0)	10 (25.0)	
Neutral, n (%)	13 (32.5)	15 (37.5)	
Dissatisfied, n (%)	1 (2.5)	12 (30.0)	
Very dissatisfied, n (%)	0 (0.0)	1 (2.5)	

Bold values indicate statistically significant results (*P* < .05).

* Mann–Whitney *U* test.

## 4. Discussion

This study investigated the effects of nicardipine and esmolol on hemodynamic control, renal function, and regional renal oxygenation in patients undergoing CH during rhinoplasty. Renal NIRS was employed to monitor regional renal oxygenation, and renal function was assessed using serum creatinine and urea levels. According to our findings, esmolol was associated with lower intraoperative bleeding, reduced additional opioid requirements, and higher surgical satisfaction than nicardipine. Repeated-measures analysis demonstrated significant time-dependent changes in MAP and renal rSO_2_ values. The MAP pattern differed significantly between groups, whereas left renal rSO_2_ showed both a significant group effect and a significant time × group interaction. Right renal rSO_2_ showed a significant time × group interaction without a significant overall group effect.

As our study utilized a short-duration surgical model, with an average surgical time of approximately 60 to 70 minutes and CH limited to 60 minutes, the findings regarding renal outcomes should be interpreted as exploratory. However, it is important to note that patients undergoing specific aesthetic procedures such as rhinoplasty, in which CH is commonly applied, generally represent an inherently low-risk population. Therefore, our study cohort reflects the typical clinical demographics of this type of surgery.

Achieving and maintaining a bloodless surgical field is of great importance in rhinoplasty because intraoperative bleeding can significantly affect surgical precision and visualization. In this context, CH has been the focus of scientific and clinical interest for several years. Many studies have demonstrated that CH provides improved surgical visualization and a better operative field.^[7,8]^ Although the efficacy of nicardipine^[20]^ and esmolol^[10]^ has been previously evaluated, direct comparisons between these agents remain limited. In our study, patients in the esmolol group exhibited less intraoperative bleeding than those in the nicardipine group. This observation may be attributed to the β-blocker mechanism of esmolol, which reduces heart rate and cardiac output and may thereby contribute to reduced bleeding in the surgical field.^[21]^

Erbesler et al^[11]^ demonstrated that esmolol provides excellent surgical field visibility and high surgeon satisfaction during CH, comparable to potent α_2_ agonists. Building upon this, our study found that esmolol yielded significantly higher surgical satisfaction scores than nicardipine, likely because of its superior ability to minimize surgical bleeding. Furthermore, intraoperative β-blockers are known to decrease supplemental opioid requirements.^[22,23]^ In a study by Erbesler et al^[11]^ only 8% of patients receiving esmolol required additional intraoperative analgesia. This aligns with our findings, where only 20% of the esmolol group required additional opioids compared with 87.5% of the nicardipine group, further supporting the opioid-sparing profile of esmolol during CH.

Regarding renal function, our observation of a postoperative decrease in serum creatinine levels contrasts with some previous studies. Guney et al^[12]^ reported a statistically significant postoperative increase in creatinine levels following CH during nasal surgery, although the values remained within the normal clinical range. Similarly, Park et al^[24]^ investigated CH during spinal surgery with considerably longer hypotensive periods, averaging 139 to 141 minutes. Although they noted that overall renal function was preserved, several patients met the RIFLE criteria for acute kidney injury due to serum creatinine elevations exceeding 1.5 times the baseline. In contrast, although our study demonstrated a postoperative decrease in creatinine levels in both groups, these values remained within normal clinical limits. This discrepancy may be primarily attributed to the shorter surgical duration and the dilutional or protective effects of perioperative hydration. Furthermore, traditional markers such as serum creatinine and urea are relatively insensitive for detecting early or subtle renal tubular injury. Additionally, our follow-up was limited to the immediate postoperative period. Therefore, the unchanged serum creatinine and urea levels observed in our study should be interpreted cautiously and cannot be considered definitive evidence of complete renal safety or the absence of subclinical acute kidney injury.

Consistent with prior clinical investigations evaluating CH,^[10–12]^ our study demonstrated that both agents achieved controlled reductions in MAP. Repeated-measures ANOVA showed a significant time effect and a significant time × group interaction for MAP, indicating that MAP changed over time and that the temporal MAP pattern differed between groups. However, the overall group effect for MAP was not significant, suggesting that neither agent produced a consistently lower MAP across the entire intraoperative period.

Regarding renal tissue oxygenation, no clinically significant renal desaturation event, defined as a ≥20% decrease from baseline renal rSO_2_, was detected in either group. For right renal rSO_2_, repeated-measures analysis showed a significant time effect and a significant time × group interaction, but no significant overall group effect. For left renal rSO_2_, significant time, group, and time × group effects were observed, indicating that left renal rSO_2_ was overall higher in the nicardipine group and followed a different temporal pattern between groups. This difference may be related to the distinct pharmacological profile of calcium channel blockers. Nicardipine exerts direct vasodilatory effects on the renal vasculature, potentially enhancing regional microcirculatory oxygenation.^[9]^ In contrast, β-blockers primarily lower systemic blood pressure by decreasing heart rate and cardiac output, which may influence regional oxygen delivery through a different physiological pathway. Nevertheless, it is crucial to emphasize that NIRS technology reflects regional, venous-weighted tissue oxygen saturation and does not directly quantify absolute renal blood flow, glomerular filtration rate, or functional renal protection. Therefore, the higher renal rSO_2_ values observed with nicardipine should be interpreted as exploratory physiological findings rather than evidence of renal protection.

### 4.1. Limitations

This study has several limitations. First, CH was limited to 60 minutes in a short-duration, low-risk population. Therefore, our findings cannot be directly extrapolated to high-risk patients or prolonged hypotensive procedures. Second, renal function was assessed only using serum creatinine and urea in the immediate postoperative period. Because these markers are relatively insensitive for early or subclinical acute kidney injury, delayed or subtle renal tubular injury cannot be definitively excluded. Future studies should include longer follow-up and more sensitive renal biomarkers, such as cystatin C, NGAL, or KIM-1. Third, renal NIRS remains an indirect measure of regional tissue oxygenation and may be influenced by overlying skeletal muscle and subcutaneous tissue. Therefore, our renal NIRS findings should be interpreted as exploratory regional oxygenation data rather than direct evidence of renal perfusion, glomerular filtration, or functional renal protection. Finally, although the revised sample size estimation was based on adult renal NIRS studies, validated renal NIRS thresholds during CH in rhinoplasty remain unavailable, and the study may have been underpowered to detect subtle renal oxygenation differences or delayed renal outcomes.

## 5. Conclusion

In conclusion, in this short-duration, low-risk rhinoplasty model using controlled hypotension, esmolol provided more favorable hemodynamic stability, reduced bleeding and opioid use, and yielded higher surgical satisfaction than nicardipine. Neither agent significantly altered standard renal function markers. Although nicardipine was associated with higher rSO2, likely owing to its direct vasodilatory effects, this finding should be interpreted as an exploratory physiological observation rather than evidence of functional renal protection. Further studies involving high-risk patients, prolonged hypotension, and sensitive renal biomarkers are necessary to clarify the renal implications of different hypotensive agents during controlled hypotension.

## Acknowledgments

The authors thank Prof Siddik Keskin, MD (Department of Biostatistics), for his expert statistical consultation and assistance with the statistical analysis. Written permission was obtained from Prof Siddik Keskin to be named in the Acknowledgments section.

## Author contributions

**Conceptualization:** Kamil Cintan, Nureddin Yüzkat, Canser Yilmaz Demir, Yunus Emre Tunçdemir.

**Data curation:** Nureddin Yüzkat, Canser Yilmaz Demir.

**Formal analysis:** Kamil Cintan, Nureddin Yüzkat.

**Investigation:** Nureddin Yüzkat.

**Methodology:** Kamil Cintan, Nureddin Yüzkat, Yunus Emre Tunçdemir.

**Software:** Kamil Cintan, Nureddin Yüzkat.

**Supervision:** Canser Yilmaz Demir, Yunus Emre Tunçdemir.

**Validation:** Canser Yilmaz Demir, Yunus Emre Tunçdemir.

**Writing – original draft:** Kamil Cintan, Nureddin Yüzkat, Canser Yilmaz Demir, Yunus Emre Tunçdemir.

**Writing – review & editing:** Kamil Cintan, Nureddin Yüzkat, Canser Yilmaz Demir, Yunus Emre Tunçdemir.
